# Complementary Medicine: Risks to Infants and Children

**DOI:** 10.3390/jcm7060149

**Published:** 2018-06-12

**Authors:** Terry Polevoy

**Affiliations:** 33-285 Sandowne Dr., Waterloo, ON N2K 2C1, Canada; drpolevoy@yahoo.com; Tel.: +1-519-574-0550

**Keywords:** complementary alternative medicine, quackery, infants, children, regulation, anti-vaccine, autism, cancer, chiropractic, naturopathy, homeopathy, health fraud

## Abstract

Complementary Alternative Medicine (CAM) that targets infants and children can place them at grave risk. Our plan is to review some of the major cases of CAM used by parents, and by unlicensed and even licensed health professionals. Complications from CAM are rarely the focus of regulatory bodies, or even the courts. Even regulated health professionals, who have profited by misrepresenting science-based evidence for treatment or prevention of disease, have been rarely sanctioned by their respective Boards or Colleges. This must change. In addition, there is a dire need for regulators, including the governments, who allow quack products and devices to be marketed in their respective countries, to prosecute them. Existing regulations must be coupled with more effective enforcement. Some of these cases have had a direct connection to me personally, while others are examples of just plain dangerous quackery.

## 1. Why Does Complementary Alternative Medicine (CAM) Seem to Thrive and Why Is It Almost Always Ignored by Regulators and the Government?

Misinformation about CAM for infants and children is just far too easy to find. Just search on Amazon for “pediatrics alternative medicine” and you can find hundreds of books that ignore science-based methods. The Internet is full of so-called “experts”, including celebrities, hucksters, politicians who are in the pockets of the nutraceutical manufacturers, and yes, even licensed health professionals. 

We will review some extreme CAM cases that have resulted in fatalities. This will include some deranged parental belief systems, which directly resulted in their child’s death, with or without direct intervention by health professionals. Other cases directly involved licensed medical practitioners, who ignored everything they learned in medical school and residency training to flog their treatments, or lack of treatments (i.e., immunizations) on vulnerable families. 

Some of the topics we will review include the CAM anti-vaccine movement, the quack treatment of children with cancer, cerebral palsy, and other chronic conditions. Extreme nutritional advice by parents has also killed children. 

Here in Canada, where I trained in pediatrics, we have had our share of headline cases over the years. Some have left a deep impact on me personally, while others have stayed in the headlines for years. Others have been too often just forgotten. By writing this article, I hope that we will all be reminded of the risks when CAM endangers our children.

One of the major dangers to child health has been the regulators themselves. Medical, chiropractic, and naturopathic Boards and Colleges around the world have been lax in overseeing their members. Doctors of Medicine (MDs) have maintained their licenses, despite their opposition to immunizations. Some have promised parents that they can treat children with profound conditions, like mental retardation, cerebral palsy, and cancer. Even after numerous complaints, many practitioners have survived unscathed. 

CAM professionals may or may not actually believe what they do. Some are just snake-oil salespeople, while others are gurus who spend their lives mesmerizing their flock of true believers. 

## 2. Chiropractic Pediatrics

Chiropractors who treat infants, children, and even pregnant mothers have formed professional associations like the International Chiropractic Pediatrics Association (ICPA). In my opinion, they exist to sustain the unbelievable charade that infants and children should go to a chiropractor from birth [1]. This group claims to be experts. But, in fact all they do is to promote parental fear that their babies have been “damaged” when they go through the birth canal. They make bogus claims that this will result in serious health problems later on in life [2]. Marketing organizations are widespread, selling full-scripts, supportive flyers, magazines, web sites, and videos that target the vulnerable minds of parents [3].

A simple search of pediatric chiropractic web sites reveals most of their false claims. The trouble is that chiropractic regulatory bodies refuse to take any firm action against their members who violate their Standards or Policies. In 2013, a group of four chiropractic authors from Canada published a review of Vaccinations and Chiropractic [4].

In Ontario, the College of Chiropractors of Ontario (CCO) has a history of sitting on complaints for years, and then when complaints are appealed to our *Health Professions Appeal and Review Board (HPARB)*, and are sent back to the CCO, it again takes years for the CCO to respond. 

One of the most egregious examples of the CCO’s lack of firm action goes way back to the 1990s, when many of their members were known for anti-vaccine activities. It took years for them to pass any meaningful policies or standards to control it. 

A prime example that I personally witnessed in 1997 here in Waterloo, Ontario, Canada, involved Jeffrey Winchester, DC. He placed himself in front of the *Bluevale Collegiate Institute*, our local high school, and told the students that they really did not have to get the meningitis vaccine. Two teenage girls had just died, but it did not stop his bizarre protest. I have many examples in my collection of anti-vaccine chiropractors here in Ontario, but very few of them were adjudicated.

Chiropractic promotions at health fairs, malls, and in the media, are sometimes more like a “Three Ring Circus”. Over the years, I have attended a number of large and small presentations where chiropractors set up shop and try to sell various programs. Some of them targeted families with young children. One of the funniest was Dr. Roger Turner, a DC from Barrie, Ontario. He and his young son, who was not a chiropractor, claimed to be able to move the skull bones to improve kids with autistic spectrum disorder (ASD). I even volunteered to have my skull manipulated at a quack-filled alternative health convention in Toronto. 

In May 2018, Sharon Kirkey, a reporter for Canada’s National Post newspaper, authored one of the best articles on pediatric chiropractors [5]. It was met with applause from the usual science-based groups and authorities [6]. 

There is absolutely no debate that those who adhere to their title of “pediatric chiropractor” really love their stuff. In September 2018, one group organized the *Chiropractic Pediatric & Family Conference* in Newark, NJ, USA [7]. The usual anti-vax brigade will be there along with the usual sales people selling all sorts of goodies to help chiropractors build their practices. Among the speakers are Canadian and American chiropractors, along with a long history of anti-medical opinions. They have even invited Larry Palevsky, an anti-vaccine MD from NYC to round out the conference. 

In Summary, the basic spin of pediatric chiropractors has never been confirmed in any major study. But, the case against them has not seemed to stop their marketing in any way. As more chiropractors graduate each year, they are having difficulty finding practice opportunities that would help them pay back their tuition. That is my belief! That is why there are so many of them who have swallowed the sales pitch of organizations who skillfully sell them expensive programs.

## 3. Dangers of Licensed and Unlicensed Quacks

There are thousands of unlicensed individuals who prey on children and their families around the world. In developing countries, they are called shamans, or “medicine men” or “medicine women”. I will not be discussing this. But, I will attempt to identify an area of grave concern, especially when it comes to children in Canada.

Children are victimized because either their parents allow them to be treated by these quacks, instead of real medical professionals, or their health providers, who are licensed as regulated health professionals, treat them without regard for science-based medicine. 

I will choose from a few of these tragic cases here in Canada, where even though we have “socialized medicine”, the practice of quackery sadly exists. Little has changed over the last few decades. It is particularly troublesome here in Ontario, because we have authorized the regulation of naturopathy, homeopathy, and traditional Chinese medicine. Socialized medicine was supposed to provide oversight of these CAM professions, but we have found little has changed. Claims made by these “professionals” have little basis in science, and some are downright dangerous. Fortunately for us, very few of their flock try to recruit infants and children…yet!

### CAM Tragedies

*Ravi Devgan*: Nearly two decades ago, I was contacted by a reporter in Ontario who had identified a poor Mexican Mennonite family who had twins that had major neurologic and mental health issues. They reached out to a physician, Ravi Devgan, who claimed to be able to cure them. Using dangerous injections of sheep fetuses, he defrauded them for over $30,000 [8].

Devgan was sentenced to jail. But, even after his first conviction and the loss of his license, he continued to rip people off from his house in Toronto. He died in prison after his last conviction.

*Tyrell Duec*: In the spring of 1999, a 13-year-old Saskatchewan boy, Tyrell Dueck garnered headlines around the world because his bone cancer treatment was stopped by his parents. They were convinced by a local chiropractor, lawyer, who was a fundamentalist Christian, and politician that the best treatment was to pray and take him to a quack clinic in Tijuana for treatment. He died of his cancer in July 1999 after the courts said that the parents could basically do what they wanted. His death raised many issues, ethically, morally, and in the courts; it was the death of Tyrell Dueck that sent me on my journey of “quackbusting” [9,10,11].

*Makayla Sault*: An 11-year-old girl from the Credit First Nation Reserve in Caldonia, Ontario, became the victim of Brian Clement. He is a notorious Florida-based quack, who runs the *Hippocrates Health Institute*, a facility in West Palm Beach, Florida. The case pitted the McMaster University Pediatric Oncology Department, social service agencies, and the First Nation’s chief against each other. The courts finally gave the go-ahead to stop chemotherapy for her leukemia, which had a good chance of putting her into remission. 

The case involved more than one Aboriginal girl suffering from treatable leukemia. The State of Florida had originally charged Clement with practicing medicine without a license. The case was quietly dropped in March 2015. One of the puzzling things about Clement is the question of his supplying illegal supplements, which were shipped to the Reserve and other health food stores in Ontario. This was reported to Health Canada, but little seemed to be accomplished. Furthermore, the Aboriginal band sponsored several appearances of Clement to recruit more patients and flog his quackery. Clement continues to flog nonsense treatments and prey on vulnerable people. We can assume that there are many more cases like Makayla in his files [12,13,14,15,16,17,18].

*Ezekiel Stephan*: This case has been in the headlines for six years. It began when a 19-month-old toddler from Alberta died of meningitis. Years ago, thousands of infants and toddlers died of meningitis in North America every year. What makes the 2012 death of Ezekiel especially unusual was the legal case against his parents. His father, David Stephan, and mother, Collet, were charged and convicted in an Alberta Court for failing to provide the necessaries of life in 2016. After years of legal battles and appeals, the case finally reached Canada’s Supreme Court in May 2018, where the case was overturned on a technicality. David and Collet have to be retried all over again back in Alberta. 

There have been scores of incisive articles, extensive television coverage, and ethical and legal opinions from around the world. The case took years to finally be prosecuted, and the actual trial lasted for weeks. Public opinion, which was largely against the couple from the start, seemed to be opposed due to David Stephan’s blogs, television appearances, and especially his own Facebook pages which were basically his way of casting the blame on others. He has never, to this day, told the public that he or his wife had any role in Ezekiel’s horrible death. 

The most interesting part of the case was David Stephan’s direct links to *Truehope Nutritional Support*, a company started by his father Anthony Stephan. It markets natural health products and has withstood Health Canada’s challenges for years. David works for the company directly. The company’s early days was the focus of our book, *Pig Pills, Inc*. *The Anatomy of an Academic and Alternative Health Fraud* [19]. The book was co-authored with Marvin Ross and Ron Reinhold. 

Truehope’s original product, *EmpowerPlus*, made major claims for treating severe mental illness. They marketed it from Alberta, but it has always been made by several companies in the U.S. The reason I mention this company is because the Stephans used them to treat their own children, particularly Ezekiel. 

The foundation for the charges and the conviction were because the parents failed to provide the necessaries of life. During the trial, it was revealed that Ezekiel had never been to a medical doctor. He was delivered at home in rural Alberta by a nurse. Ezekiel, like his other siblings, had never received any immunizations. 

When Ezekiel became ill with a respiratory condition on 27 February 2012, his health went downhill steadily. Even after he was visited by his grandfather, Anthony Stephan, there was no improvement. He became so “stiff” that he was unable to get into his car seat. He was finally rushed to the *Calgary Children’s Hospital* in a coma and was declared “brain dead” on 12 March 2012. The post-mortem showed bacterial meningitis and an empyema (pus in the lung). 

Why did the parents fail to recognize how sick he was? Were they entirely to blame? They clearly ignored warning signs that any parent should have recognized. Why did Collet take him to see a naturopath in Lethbridge, but never brought him into the office? It seemed that she just asked the naturopath’s front office for *BLAST*, an natural health product containing echinacea. 

Of course, his meningitis could have easily been prevented and his life could have been saved if they were caring parents. Instead, they were basically anti-vaccine, anti-medical zealots who instead treated him with olive leaf extract, garlic, onions, pepper, ginger root, and horseradish. 

The Crown’s evidence was overwhelming, and throughout the trial, David would rant and rave of how bad the Government’s case was. It was a giant conspiracy! Evidence was tampered with or withheld! The pathologist was wrong! There was a massive cover-up!

After their conviction by a jury in April 2016, they appealed. The appeal was finally heard in November 2017. David Stephan continued to attack the original conviction with insane rants against the prosecution’s case and the jury’s decision. He concocted a conspiracy theory about withheld evidence, that Ezekiel did not actually have meningitis or an empyema, and that the ambulance drivers and the staff of the Children’s Hospital caused Ezekiel’s death because they didn’t have the right sized tube when they attempted to intubate him during transport to Calgary. 

In May 2018, the case was thrown out by the Supreme Court of Canada in Ottawa. The court ordered a new trial on the original charges. If the Stephans were again convicted, they might actually face more severe consequences.

## 4. Lessons Learned?

What can we learn from the dangers of CAM and the risks to our children?

- CAM practitioners are sometimes just basically crooks and frauds who have no morals.
- Regulated health professionals who are trained to believe that they have the answers, when they demonstrate no evidence that their treatments works.
- Parents who ignore basic public health measures.
